# Variability of the Posterior Tibial Slope in Saudis: A Radiographic Study

**DOI:** 10.7759/cureus.10699

**Published:** 2020-09-28

**Authors:** Wazzan ALJuhani, Salman S Qasim, Mohammed Alsalman

**Affiliations:** 1 Surgery, King Abdulaziz Medical City in Riyadh, Riyadh, SAU; 2 Medicine, King Saud bin Abdulaziz University for Health Sciences and King Abdullah International Medical Research Center, Riyadh, SAU; 3 Medicine, King Saud bin Abdulaziz University for Health Sciences, Riyadh, SAU; 4 Radiology, King Abdulaziz Medical City in Riyadh, Riyadh, SAU

**Keywords:** knee, plain x-rays, lateral knee radiograph, posterior tibial slope

## Abstract

Aim

The present study aimed to establish the normal range of the posterior tibial slope (PTS) angle in the Saudi adult population and to identify whether there was an association between the angle and gender or age.

Materials and methods

A total of 524 normal knee radiographs of 410 patients aged 18-85 years were included in the study. The PTS was measured using the anterior tibial cortex method. Data were matched with gender and age for statistical analysis.

Results

The mean physiological PTS angle was 13.6 ± 3.4˚ (range: 3.8-23.9˚). Age and gender did not influence the PTS value (P >0.05). The two-way analysis of variance (ANOVA) test showed no interaction effect between age and gender on the PTS (P >0.05).

Conclusions

This study provided a reference range for the normal PTS among Saudis, which can assist in decision-making during different knee procedures. The PTS value did not significantly differ between male and female subjects, and there was no significant association between the PTS angle and age.

## Introduction

The posterior tibial slope (PTS) is defined as the angle between the longitudinal axis of the tibia and the posterior inclination of the tibial plateau [1]. The PTS plays a significant role in knee joint stability and biomechanics [2], and its increase is associated with an increased risk of developing various medical conditions. For example, there is an increased risk of anterior cruciate ligament (ACL) tear in the presence of an increased lateral PTS, which indicates a positive correlation between PTS angle and ACL injury [3,4]. Patients with increased medial or lateral PTS have an increased risk of developing anterior tibial translation (ATT) [5]. Thus, PTS correlates positively with ATT and is considered a risk factor for ATT. Furthermore, it has been proven that a steeper or excessive PTS angle leads to progressive loosening of the tibiofibular joint gap secondary to decreased collateral ligament tension during knee flexion [6].

A reduced PTS angle, on the other hand, increases posterior cruciate ligament (PCL) strain during total knee arthroplasty and may be considered a contributing factor in failed PCL reconstruction surgeries [7,8]. The PTS angle is also considered during the management of other clinical conditions, such as opening-wedge high tibial osteotomy and early ACL reconstruction in cases of ACL-deficient knees [9,10]. Consequently, the surgical decisions and outcomes of knee arthroplasty are significantly influenced by alterations in the PTS angle.

The standard value of the PTS significantly varies according to the reference point and imaging modalities [11,12]. Studies conducted among various ethnic groups show differing normal values for PTS. For instance, the mean normal PTS angle among the adult Igbo population of South East Nigeria was found to be 11.9˚ [13], while it was found to be 14.7˚ among the Chinese [14], and 14.1˚ and 12.5˚ among female and male Pakistani adults, respectively [15].

Since many studies have demonstrated PTS angle differences that are relative to ethnicity and gender, and given the lack of recorded values for the physiological PTS angle among the Saudi population, the primary aim of this study was to establish a normal range for the same. This would enable surgeons to maintain appropriate PTS values during knee surgery as well as serve as a decision-making tool during surgery. The secondary objectives of our study were to determine any significant difference in PTS between genders and to identify a potential association between the angle and age.

## Materials and methods

This retrospective cohort study was conducted at King Abdulaziz Medical City (KAMC) in Riyadh, Saudi Arabia. A total of 7157 knee radiographs of adult Saudi patients at KAMC between August 1, 2019, and December 1, 2019, were reviewed using the Centricity Enterprise (GE Healthcare Pvt Ltd, Piscataway, NJ) picture archiving and communication system (PACS). From these, 524 knee radiographs taken from 410 patients aged 18-85 years were included in the study. The inclusion criterion was patients aged at least 18 years who had taken plain X-rays of the knee that the radiologist reported as normal. Exclusion criteria were X-rays of non-Saudi patients or suboptimal X-rays of the knee, patients with an immature skeleton, radiologically proven knee pathologies, such as fractures, osteoarthritis, or tumors, or previous knee surgery or implant.

**Figure 1:**
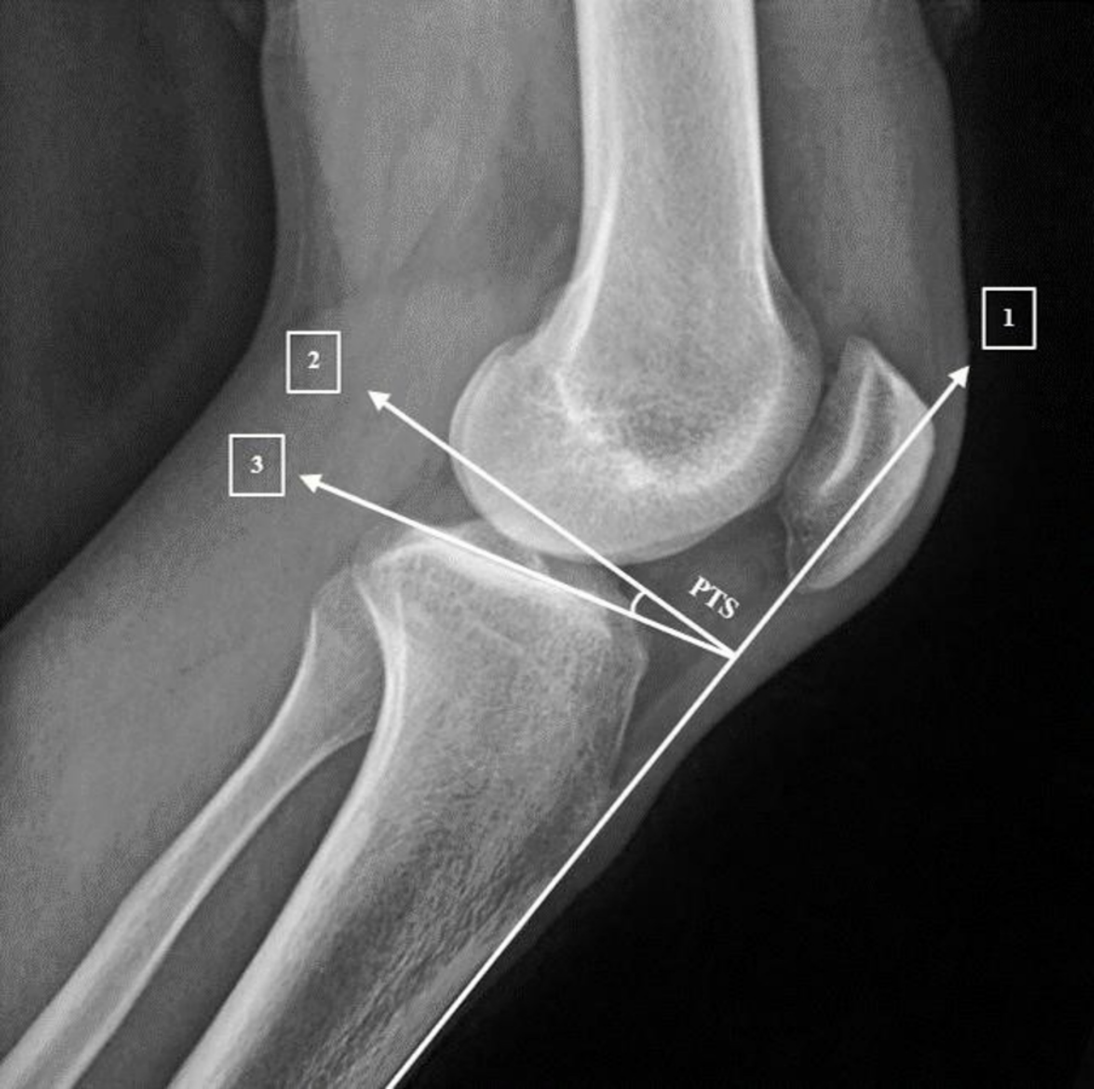
PTS measurement using the anterior tibial cortex method. PTS: posterior tibial slope.

For angle measurement, true lateral X-rays of the knee with femoral condyles superimposed were viewed using the GE Centricity PACS software. A straight line was drawn tangential to the anterior tibial cortex (ATC) representing the tibial axis. A second line was drawn perpendicular to line one. A third line was drawn along the tibial plateau. The angle formed by line two and line three represents the PTS angle (Figure 1).

To ensure anonymity, serial numbers were used instead of medical record numbers. All the data were recorded on a protected Excel spreadsheet (Microsoft Corporation, Redmond, WA) and were only used by research members. Ethical approval was obtained from the institutional review board and the requirement for informed consent was waived due to the use of anonymized patient data and the retrospective nature of the study. Patients' medical records were accessed through the BESTCare system to obtain patients’ demographic data. The GE Centricity PACS software was used for the measurement of the PTS angle.

The PTS angle measurements were all taken by a single observer under the guidance and supervision of an experienced consultant musculoskeletal radiologist. A total of 40 randomly selected lateral knee radiographs were measured twice by the single observer, four weeks apart. The intraclass correlation was used to estimate the intraobserver reliability. The intraclass correlation coefficient (ICC) was found to be 0.88, which represents a strong agreement between the two readings.

The data analysis process of this study consisted of two stages. The first stage included a descriptive analysis where numerical variables were reported in terms of means and SDs. The second stage included data analysis using the independent samples t-test to compare the PTS value between males and females, the one-way analysis of variance (ANOVA) test to compare the PTS value between age groups, and the two-way ANOVA test to examine the influence of age and gender on the PTS value, which were applied using IBM SPSS Statistics 25.0 (IBM, Armonk, NY).

## Results

Of the included participants, 159 (39%) were males and 251 (61%) were females. Participants were grouped into three age categories: young adult (18-40 years), middle-aged (41-64 years), and elderly (65 years and above) (Table 1). A total of 279 (53%) radiographs were of the right knee, and 245 (47%) were of the left knee with a PTS mean of 13.55 ± 3.38˚ (range: 4.3-23.9˚) and 13.68 ± 3.25˚ (range: 3.8-21.0˚) for the right and left knees, respectively (Table 2). The independent samples t-test showed no significant difference in PTS angle between males and females in both the right and left knees (P >0.05) (Table 3). The one-way ANOVA test indicated no significant difference in PTS angle between age groups (P >0.05) (Table 4). The two-way ANOVA test showed no interaction effect between age and gender on PTS value (P >0.05) (Table 5). 

**Table 1:** Demographics

Characteristics	N (%)
Age, years	
Young adult (18-40)	275 (67.1)
Middle-age (41-64)	124 (30.2)
Elderly (65 and above)	11 (2.7)
Gender	
Male	159 (38.8)
Female	251 (61.2)

**Table 2:** PTS (°) mean values, SDs, and ranges for the left and right knees LK: left knee; PTS: posterior tibial slope; RK: right knee; SD: standard deviation

	Mean	SD	Range	Overall mean ± SD
PTS RK (n = 279)	13.6	3.4	4.3-23.9	13.6 ± 3.4
PTS LK (n = 245)	13.7	3.3	3.8-21.0	

**Table 3:** Comparison between PTS (°) mean values for the left and right knees with gender (done by independent samples t-test) LK: left knee; PTS: posterior tibial slope; RK: right knee; SD: standard deviation

	Male		Female		t-value	P-value
	Mean	SD	Mean	SD		
PTS RK	13.5	3.6	13.6	3.2	-0.37	0.714
PTS LK	13.7	3.7	13.7	3	-0.09	0.928

**Table 4:** Comparison between PTS (°) mean values for the left and right knees with age (done by one-way ANOVA test) ANOVA: analysis of variance; LK: left knee; PTS: posterior tibial slope; RK: right knee; SD: standard deviation

	Young adult (18-40 years)		Middle-age (41-64 years)		Elderly (65 years and above)		F-score	P-value
	Mean	SD	Mean	SD	Mean	SD		
PTS RK	13.5	3.3	13.8	3.6	12	2.6	0.97	0.382
PTS LK	14	3.2	13.3	3.2	12.4	4.6	1.68	0.189

**Table 5:** Comparison between PTS (°) mean values for the left and right knees with age and gender (done by two-way ANOVA test) ANOVA: analysis of variance; LK: left knee; PTS: posterior tibial slope; RK: right knee

Variables	Male			Female			F-score			P-value		
Age	Young adult (18-40 years)	Middle-age (41-64 years)	Elderly (65 years and above)	Young adult (18-40 years)	Middle-age (41-64 years)	Elderly (65 years and above)	Gender	Age	Gender * age (interaction effect)	Gender	Age	Gender * age (interaction effect)
PTS RK	13.4 ± 3.7	13.9 ± 3.7	12.6 ± 2.8	13.5 ± 3.0	13.8 ± 3.5	10.8 ± 2.1	0.35	1.11	0.26	0.554	0.331	0.771
PTS LK	14.0 ± 3.5	12.8 ± 3.7	12.9 ± 5.9	13.9 ± 2.9	13.5 ± 3.1	11.4 ± 1.1	0.16	2.16	0.58	0.688	0.118	0.560

## Discussion

PTS plays a crucial role in maintaining the stability of the knee [2]. Many studies have demonstrated a positive correlation between PTS and ATT, increasing the risk of ACL injury [16]. Several reference points, such as the posterior or anterior tibial cortex, tibial proximal anatomical axis, fibular shaft axis, and fibular proximal anatomical axis, can be used to represent the tibial axis [17-20]. It was reported that the PTS can vary significantly according to the reference axis used in the measurement [21]. In our study, measurement of the PTS angle was done with the ATC method, which was first introduced by Moore and Harvey [22]. The line drawn tangential to the ATC was used as a reference point because it was commonly used by surgeons to align the cutting jig during total knee arthroplasty [14].

Differences in the PTS among ethnic groups have already been reported [13-15,20,23]. Table 6 presents PTS angles measurements among different populations, using a similar reference axis (ATC). The findings in our study demonstrate a mean of 13.6 ± 3.4˚ for the PTS in a section of the Saudi population. There was no association between PTS angle and age in the studied sample (P >0.05). This finding is similar to that of Khattak et al. [15] and Marouane et al. [16]. In contrast, a study by Sun et al. [24] conducted among the Chinese found that the PTS showed a trend of first decreasing then increasing with advancing age.

**Table 6:** PTS values in different populations (measured using the ATC method) ATC: anterior tibial cortex; PTS: posterior tibial slope; SD: standard deviation

Authors	Population	Gender	Sample size (knee radiographs)	PTS (˚)	
				Range	(Mean ± SD)
Katchy et al. [13]	Nigerian (Igbo)	Male and female	530	1-25	11.9 ± 3.4
Chiu et al. [14]	Chinese	Male and female	50	5-22	14.7 ± 3.6
Khattak et al. [15]	Pakistani	Male and female	118	-	13.3 ± 4.1
Yoo et al. [20]	Korean	Female	90	5-23.2	13.8 ± 3.5
Medda et al. [23]	Indian	Male and female	184	6-24	13.6 ± 3.5
Present study	Saudi	Male and female	524	3.8-23.9	13.6 ± 3.4

During the literature review, we found contrasting findings regarding the association between PTS and gender. In the present study, there was no significant difference in PTS between males and females (P >0.05). Likewise, Katchy et al. [13] and Medda et al. [23] found no sexual dimorphism among Nigerian and Indian subsets, respectively. MRI of the knee is the gold standard modality by which medial and lateral tibial plateaus can be assessed separately [19]. Hashemi et al. [25] measured the medial and lateral PTS angles with MRI and found that in females both the medial and lateral slopes were significantly larger than in males. It is worth noting that Hashemi et al. [25] used the tibial anatomical axis as a reference point for their measurements; meanwhile, the reference axis used in the present study was the ATC. Khattak et al. [15] evaluated the medial and lateral tibial plateaus separately in healthy Pakistani volunteers of both genders using true lateral knee radiographs, similar to the method in our study. In their study, the medial PTS in females was significantly greater than in males, while the lateral PTS showed no significant difference between genders. There were some limitations to the present study. The PTS of the medial and lateral tibial plateau could not be measured separately; this is due to the measurements being taken from conventional two-dimensional (2D) radiographs. Measurements of PTS in this study were only taken using the ATC method. Various methods for measuring the posterior slope angle have been introduced [21], which may produce a remarkable difference in the PTS value. However, values obtained using the six different methods maintained a strong correlation [21].

Since the main aim of this study was to determine the normal PTS angle in Saudis, only normal knee radiographs were included. Radiographs with any pathological features, such as osteoarthritic changes or fractures, or previous knee surgery that could alter tibial anatomy or suboptimal radiographs of the knee, were excluded from the study, thereby reducing the influence of possible confounders. Radiographs in the study were true lateral X-rays of the knee with femoral condyles superimposed, which produce more accurate PTS measurements.

Our findings are clinically crucial for Saudis undergoing knee surgeries, such as total knee arthroplasty, tibial plateau reconstruction, and ACL repair, as the PTS value serves as a decision-making tool in these surgeries. Studies have shown that individuals with an excessive PTS angle have an increased risk of developing ACL injury [3,4,26]. This study may assist surgeons in identifying patients who are at increased risk of ACL injury. In addition to the influence of the PTS on ACL, PCL tension and knee stability are largely affected by the PTS after total knee arthroplasty [2,6]. Postoperative stiffness, abnormal femoral rollback, and polyethylene wear can be a consequence of a reduced PTS angle [6].

Establishing a normal range for PTS enables surgeons to maintain appropriate PTS values during knee surgery for better clinical outcomes. Knowledge of the presented measurements of the PTS may be beneficial in appropriate restoration of normal knee biomechanics during total knee arthroplasty, in addition to preoperative planning [13]. Knee prosthesis manufacturers are encouraged to consider the PTS measurements in the present study for the production of prostheses for people of Saudi Arabian descent.

## Conclusions

The present study provided a reference range for the physiological values of PTS in a Saudi population with healthy knees. It can be used in different orthopedic procedures involving the knee joint. There was no association between the PTS angle and age. The PTS value did not significantly differ between males and females.

## References

[1] Giffin JR, Vogrin TM, Zantop T, Woo SLY, Harner CD (2004). Effects of increasing tibial slope on the biomechanics of the knee. Am J Sports Med.

[2] Ahmad R, Patel A, Mandalia V, Toms A (2016). Posterior tibial slope: effect on, and interaction with, knee kinematics. JBJS Rev.

[3] Webb JM, Salmon LJ, Leclerc E, Pinczewski LA, Roe JP (2013). Posterior tibial slope and further anterior cruciate ligament injuries in the anterior cruciate ligament-reconstructed patient. Am J Sports Med.

[4] Bojicic KM, Beaulieu ML, Imaizumi Krieger DY, Ashton-Miller JA, Wojtys EM (2017). Association between lateral posterior tibial slope, body mass index, and ACL injury risk. Orthop J Sports Med.

[5] Li Y, Hong L, Feng H (2014). Posterior tibial slope influences static anterior tibial translation in anterior cruciate ligament reconstruction: a minimum 2-year follow-up study. Am J Sports Med.

[6] Kang KT, Koh YG, Son J, Kwon OR, Lee JS, Kwon SK (2018). Influence of increased posterior tibial slope in total knee arthroplasty on knee joint biomechanics: a computational simulation study. J Arthroplasty.

[7] Singerman R, Dean JC, Pagan HD, Goldberg VM (1996). Decreased posterior tibial slope increases strain in the posterior cruciate ligament following total knee arthroplasty. J Arthroplasty.

[8] Bernhardson AS, Aman ZS, DePhillipo NN (2019). Tibial slope and its effect on graft force in posterior cruciate ligament reconstructions. Am J Sports Med.

[9] Naudie DDR, Amendola A, Fowler PJ (2004). Opening wedge high tibial osteotomy for symptomatic hyperextension-varus thrust. Am J Sports Med.

[10] Lee JJ, Choi YJ, Shin KY, Choi CH (2011). Medial meniscal tears in anterior cruciate ligament-deficient knees: effects of posterior tibial slope on medial meniscal tear. Knee Surg Relat Res.

[11] Hudek R, Schmutz S, Regenfelder F, Fuchs B, Koch PP (2009). Novel measurement technique of the tibial slope on conventional MRI. Clin Orthop Relat Res.

[12] Zhang Y, Wang J, Xiao J, Zhao L, Li ZH, Yan G, Shi ZJ (2014). Measurement and comparison of tibial posterior slope angle in different methods based on three-dimensional reconstruction. Knee.

[13] Katchy AU, Njeze NR, Nevobasi IO, Nnamani K, Ata AU (2018). Posterior tibia slope angle measurement in adult Igbos of South Eastern Nigeria using plain Xray films. Int J Med Heal Dev.

[14] Chiu KY, Zhang SD, Zhang GH (2000). Posterior slope of tibial plateau in Chinese. J Arthroplasty.

[15] Khattak MJ, Umer M, Davis ET, Habib M, Ahmed M (2010). Lower-limb alignment and posterior tibial slope in Pakistanis: a radiographic study. J Orthop Surg (Hong Kong).

[16] Marouane H, Shirazi-Adl A, Adouni M, Hashemi J (2014). Steeper posterior tibial slope markedly increases ACL force in both active gait and passive knee joint under compression. J Biomech.

[17] Kim KH, Bin SI, Kim JM (2012). The correlation between posterior tibial slope and maximal angle of flexion after total knee arthroplasty. Knee Surg Relat Res.

[18] Oka S, Matsumoto T, Muratsu H (2014). The influence of the tibial slope on intra-operative soft tissue balance in cruciate-retaining and posterior-stabilized total knee arthroplasty. Knee Surg Sports Traumatol Arthrosc.

[19] Karimi E, Norouzian M, Birjandinejad A, Zandi R, Makhmalbaf H (2017). Measurement of posterior tibial slope using magnetic resonance imaging. Arch Bone Jt Surg.

[20] Yoo JH, Chang CB, Shin KS, Seong SC, Kim TK (2008). Anatomical references to assess the posterior tibial slope in total knee arthroplasty: a comparison of 5 anatomical axes. J Arthroplasty.

[21] Brazier JC, Migaud H, Gougeon F, Cotten A, Fontaine C, Duquennoy A (1996). Evaluation of methods for radiographic measurement of the tibial slope. A study of 83 healthy knees. Rev Chir Orthop Reparatrice Appar Mot.

[22] Moore TM, Harvey JP (1974). Roentgenographic measurement of tibial-plateau depression due to fracture. J Bone Joint Surg Am.

[23] Medda S, Kundu R, Sengupta S, Pal AK (2017). Anatomical variation of posterior slope of tibial plateau in adult Eastern Indian population. Indian J Orthop.

[24] Sun YH, Chen LX, Jiao ZD, Wang L, Zhang RM, Fang J, Li J (2016). Age-related changes of posterior tibial slope and its roles in anterior cruciate ligament injury. Int Surg.

[25] Hashemi J, Chandrashekar N, Gill B (2008). The geometry of the tibial plateau and its influence on the biomechanics of the tibiofemoral joint. J Bone Joint Surg Am.

[26] Wang YL, Yang T, Zeng C (2017). Association between tibial plateau slopes and anterior cruciate ligament injury: a meta-analysis. Arthroscopy.

